# Epigenome-wide DNA methylation patterns associated with disease activity in systemic lupus erythematosus

**DOI:** 10.1038/s41598-026-51708-3

**Published:** 2026-05-05

**Authors:** Amin Ravaei, Tahzeeb Fatima, Chris Wincup, Anna Rudin, Cristina Maglio, Jan Bjersing

**Affiliations:** 1 Department of Rheumatology and Inflammation Research, Institute of Medicine, Sahlgrenska Academy, University of Gothenburg, Gothenburg, Sweden; 2 Department of Microbiology and Immunology, Institute of Biomedicine, University of Gothenburg, Gothenburg, Sweden; 3 Department of Clinical and Academic Rheumatology, King’s College Hospital, London, UK; 4 Rheumatology Clinics, Sahlgrenska University Hospital, Gothenburg, Sweden

**Keywords:** SLE, DNA methylation, Epigenetics, Disease activity, Biomarkers, Computational biology and bioinformatics, Diseases, Genetics, Immunology, Neurology, Neuroscience

## Abstract

Systemic lupus erythematosus (SLE) is a complex autoimmune disease with fluctuating disease activity and heterogeneous clinical manifestations. While DNA methylation changes have been implicated in SLE pathogenesis, their relationship to disease activity remains unclear. This study aimed to identify epigenetic correlates of disease activity in women with SLE using genome-wide DNA methylation profiling. Whole blood DNA from 48 women with established SLE was analyzed using Illumina EPIC arrays. Disease activity was assessed using the Systemic Lupus Erythematosus Disease Activity Index 2000 (SLEDAI-2K). Differentially methylated positions (DMPs) and regions (DMRs) associated with disease activity were identified using linear modeling and region-level analyses, adjusting for age, ethnicity, smoking status, body mass index (BMI), and cell composition. Gene ontology enrichment and motif analyses were also performed. No individual CpGs reached FDR-corrected significance, therefore, a hypothesis-generating approach using a raw *p*-value < 0.01 was applied. Over 4,500 suggestive DMPs (*p* < 0.01) were identified and further analyzed for DMRs and pathway mapping. Thirty-six significant DMRs (at 1% false discovery rate) were detected, with several genes involved in neuroimmune regulation and systemic inflammation. Motif analysis identified REST, a transcriptional repressor that silences neuronal genes in non-neuronal tissues, as a common motif among several DMRs. Semantic analysis of the affected loci revealed five major biological domains relevant to SLE: immune signaling, neuroimmune and neural processes, organ developmental morphogenesis, metabolic regulation, and epigenetic control. This exploratory study identified potential epigenome-wide methylation changes linked to disease activity in a well-controlled cohort of women with SLE. Region-level analysis revealed DMRs mapping to immune and neuroimmune pathways and showed enrichment of the REST binding motif. Although these findings are preliminary and require independent validation, they suggest subtle epigenetic alterations that may be associated with SLE activity and points to candidate pathways for further investigation.

**Supplementary Information:**

The online version contains supplementary material available at 10.1038/s41598-026-51708-3.

## Introduction

Systemic lupus erythematosus (SLE) is a chronic autoimmune disorder with diverse clinical presentations involving a wide array of organ domains, including mucocutaneous, renal, neurological, cardiorespiratory, articular and hematological involvement^1^. The goal in SLE care is to achieve remission, which is defined as the absence of disease activity along with minimal or no use of glucocorticoids and stable doses of immunosuppressive therapy^2^. However, remission is often difficult to achieve, leading to the adoption of alternative targets such as low disease activity state^3^. To improve remission and low disease activity rates, it is crucial to investigate the biological mechanisms underlying disease activity in SLE, particularly in relation to those that remain active who may achieve low disease activity but not attain full disease remission. Furthermore, it should be considered that many patients with low disease activity continue to be troubled by persisting debilitating symptoms, such as fatigue, cognitive dysfunction, mood disturbance and non-inflammatory pain, which do not appear to reliably improve with intensification of immunosuppressive therapy^4^.

Epigenetic processes, including DNA methylation, histone modifications, and non-coding RNA regulation, modulate gene expression without altering the underlying DNA sequence^5^. Over the past two decades, accumulating evidence has underscored the role of epigenetic modifications in SLE pathogenesis, providing novel molecular insights into disease heterogeneity, sex disparities, and familial predisposition^6–8^. Among these mechanisms, DNA methylation has emerged as a central regulator, with abnormal patterns implicated both in disease development and clinical variability^9,10^. In patients with SLE, CD4^+^ T cells exhibit global DNA hypomethylation and dysregulated expression of methylation-related genes^11^. Experimental studies further demonstrate that administration of DNA methyltransferase inhibitors to activated CD4^+^ T cells induces lupus-like autoimmunity in healthy mice, whereas in mice with established lupus it ameliorates disease manifestations^12^.

Methylation profiles may vary across different SLE clinical phenotypes, activity states, and organ involvement^7,13^.Previous studies have focused on mixed or highly active cohorts when examining whether DNA methylation reflects clinical quiescence under routine treatment, defining quiescent patients as those with minimal disease activity^14^. Thus, it remains unclear whether DNA methylation can differentiate SLE patients with low-level disease activity from those in clinical remission. In this study, we investigated the association between DNA methylation alterations and disease activity scores in a well-controlled, well-characterized cohort of women with SLE, aiming to clarify the role of methylation as a potential biomarker of disease activity under treatment.

## Methods

### Study design and participants

A total of 122 individuals with SLE, recruited between 2017 and 2018 as part of the SLEGOT cohort at Sahlgrenska University Hospital in Gothenburg, Sweden, were included in this study. The diagnosis of SLE was established according to the revised 1997 American College of Rheumatology criteria^15^. From this cohort, a subgroup of 56 female patients with available clinical data (including symptoms and disease activity) and blood samples was selected.

Disease activity was assessed using the SLE Disease Activity Index 2000 (SLEDAI-2K)^16^. Patients were categorized into two groups: no disease activity (SLEDAI-2K = 0, n = 33) and evidence of ongoing detectable disease activity (SLEDAI-2K > 0, n = 15). All procedures were performed in accordance with the Declaration of Helsinki. The regional ethics committee (Dnr 198–17 and T1072-17) granted ethical approval for the study, and written informed consent was obtained from each participant prior to enrollment.

### Genome-wide DNA methylation analysis

Genomic DNA was isolated from freshly frozen whole blood samples stored at -80°C by the Bioinformatics and Expression Analysis Core Facility (BEA), Karolinska Institute, Stockholm, Sweden. The quality and quantity of isolated DNA samples were assessed using Qubit fluorometry, with DNA integrity evaluated by Agilent TapeStation analysis. All samples met the minimum quality thresholds for concentration and DNA integrity. The DNA was subjected to sodium bisulfite conversion using the Zymo EZ 96 DNA Methylation Kit (Zymo Research, Cat. No. D5004) following the manufacturer’s protocol. After conversion, the DNA samples were hybridized onto the Epic850K BeadChip and processed following the Illumina Infinium HD protocol for methylation analysis by the Bioinformatics and Expression Analysis Core Facility (BEA). Following data acquisition, the raw intensity data underwent preprocessing and quality control using the ChAMP package^17,18^ in R. During this process, low-quality or nonspecific CpG probes were excluded, and probes overlapping with single nucleotide polymorphisms (SNPs) with a minor allele frequency (MAF) greater than 1% were removed to ensure data accuracy. Methylation levels were normalized across samples using BMIQ normalization^17^, yielding β-values for subsequent analyses.

### Statistical analysis

Differences in DNA methylation between the two groups (“no disease activity” vs “detectable disease activity”) were analyzed using M-values, which were derived by applying a logit transformation to the original β-values. This transformation was performed to improve the statistical properties of the data, specifically, to stabilize variance and approximate a normal distribution, making them more suitable for linear modeling^19^. Linear models were fitted using the “limma” package^20^ in R, which supports fixed-effects modeling. Batch included as a covariate in the design matrix to adjust for potential batch effects. Additional covariates included ethnicity, age, smoking status, body mass index (BMI), and estimated cell type composition, ensuring that DNA methylation differences were evaluated independently of these potential confounders. Ethnicity was defined based on self-reported ancestry and categorized as European vs. non-European. Cell-type proportions were estimated using EpiDISH with the Robust Partial Correlations (RPC) method and a 12-cell-type EPIC reference, which models each sample’s methylation profile as a mixture of reference cell types to provide estimated proportions for major immune populations^21^. RPC is robust for large-scale, heterogeneous methylation datasets and leverages all overlapping CpGs on the EPIC 850K array. The effect of batch correction is illustrated in Supplementary Fig. 1. Estimated cell-type proportions are provided in Supplementary Table 1.

Differentially methylated CpG sites were identified using the empirical Bayes moderated t-test. To account for multiple testing and minimizing false positives, *p*-values were adjusted using the Benjamini–Hochberg procedure^22^, with a false discovery rate (FDR) threshold of *q* < 0.05 considered statistically significant.

To explore the overall structure of DNA methylation relative to disease activity, Orthogonal Partial Least Squares Discriminant Analysis (OPLS-DA) was performed using the ropls R package^23^. Analysis was conducted on the top 5,000 most variable CpG sites, selected based on variance across all samples using M-values. Data were mean-centered and scaled to unit variance prior to modeling. The model included one predictive component and was trained with fivefold cross-validation, repeated five times to ensure robustness. Model performance was evaluated using R^2^ and Q^2^ metrics, providing a quantitative summary of the variance explained by the predictive and orthogonal components.

Differentially methylated regions (DMRs) were identified using the “DMRcate” package^24^, which detects regional methylation differences across contiguous CpG sites. DMR-associated genes were defined as genes whose annotated genomic regions overlapped with identified DMRs based on the EPIC 850K array annotation. These genes were used for downstream functional enrichment analyses using “g:Profiler”^25^.

Gene set enrichment analysis on differentially methylated CpGs was performed using the “missMethyl” package^26^ to identify Gene Ontology (GO) terms associated with disease activity. GO terms with raw *p*-values < 0.05 were considered significantly enriched. Enrichment results were further analyzed using semantic similarity-based clustering to group related biological processes and overarching functional domains. Pairwise semantic similarities between GO terms were calculated using the Wang method (GOSemSim package^27^), and terms were clustered using simplifyEnrichment^28^ to identify overarching functional domains for interpretation. All statistical analyses and data visualizations were performed in R v 4.4.1 (R: The R Project for Statistical Computing).

## Results

### Study population

Of 56 patients from the SLEGOT Cohort who met the inclusion criteria, 48 were included in the final analysis, while eight were excluded due to failing quality control after processing on the EPIC 850K array using the ChAMP pipeline, defined as a high failed CpG fraction (i.e., a high proportion of probes with detection p-values > 0.01), indicating unreliable measurements. The demographic and clinical data for the analyzed cohort are summarized in Table 1. Briefly, participants had a median age of 49 years and a median disease duration of 16 years. Thirty-three participants had an SLEDAI-2K of 0 and were grouped as “No disease activity” while 15 had an SLEDAI-2K greater than 0, with a median of 3 and an interquartile range from 2 to 5 and were grouped as “Detectable disease activity”. Notably, participants in the disease activity group were younger than those in the group with no disease activity (*p* = 0.016).

**Table 1:** Characteristics of the study cohort.

Characteristics	Participants (n = 48)	Disease activity (n = 15)	No disease activity (n = 33)	*p* value
Age, years	49 (33—53)	33 (28- 50)	50 (39—55)	0.016
Ethnicity, European (%)	90	87	91	0.641
BMI, kg/m^2^	24 (22—29)	23 (21- 26)	25 (22—30)	0.083
Smoking status, Current (%)	10	13	9	0.642
Clinical features				
ESR, mm/hr ^##^	14 (5—21)	17 (5—44)	12 (6—20)	0.511
CRP, mg/L*	1 (1—2)	1 (1—5)	1 (1—1)	0.478
Disease duration, years	16 (10—26)	10 (7—20)	19 (11—27)	0.578
HAQ	0.1 (0 – 0.6)	0.1 (0 – 0.4)	0.4 (0—0.8)	0.465
EQ-5D score ^###^	0.8 (0.7—1)	0.8 (0.7 – 0.9)	0.8 (0.7 – 10)	0.889
SLICC Damage Index	0 (0—1)	0 (0—1)	0 (0—1)	0.280
Antibody				
Anti-dsDNA, ever positive (%)	56	60	55	0.969
Anti-SS-A (Ro), ever positive (%) ^###^	43	73	28	0.005
Anti-SS-B (La), ever positive (%) ^###^	23	40	16	0.141
Medication use				
Immunomodulatory drugs, using (%) **	87	87	88	0.999
Antimalarials, using (%)	67	67	67	0.999
csDMARD, using (%)	52	44	51	0.999
bDMARD, using (%)	15	27	9	0.183
Prednisolone, using (%)	42	40	42	0.987
Prednisolone > 5 mg/day, using (%)	15	20§	12§§	0.513
Prednisolone, mg	2.7 ± 3.7	2.7 ± 3.6	2.7 ± 3.8	0.940
Analgesics, using (%) ***	73	80	70	0.727
Antidepressants, using (%)	23	7	30	0.136

Values are presented as median and interquartile range (Q1–Q3). Missing data are indicated as follows: ^#^n = 42 (14 disease activity; 28 no disease activity), ^##^n = 47 (15 disease activity; 32 no disease activity), ^###^n = 44 (15 disease activity; 29 no disease activity). *Reference range: < 3 mg/L in healthy individuals. ^**^Antimalarials, conventional synthetic and biologic Disease-Modifying Anti-Rheumatic Drug (DMARD). ^***^Non-steroidal anti-inflammatory drugs (NSAIDs), paracetamol, and opioids. § 3 patients out of 15 (20%) with prednisolone dose > 5 mg/day: 2 had 7.5 mg/day and 1 had 10 mg/day. §§ 4 patients out of 33 (12%) with prednisolone dose > 5 mg/day: 1 had 7.5 mg/day, 2 had 10 mg/day and 1 had 15 mg/day. Abbreviations: BMI, body mass index; ESR, erythrocyte sedimentation rate; CRP, C-reactive protein; HAQ, Health Assessment Questionnaire; EQ-5D, EuroQol 5-Dimension score; SLICC, Systemic Lupus International Collaborating Clinics damage index; SD, standard deviation.

### Epigenome-Wide DNA methylation analysis

Following quality control and data normalization, 704,237 CpG sites were included in the analysis. Among these, 26,267 differentially methylated positions (DMPs) were identified as significant (*p* < 0.05) when comparing the disease activity group to the no disease activity group; however, none remained statistically significant after FDR adjustment (Additional file, Supplementary Table 2). To reduce false positives, 4,542 DMPs with a raw *p*-value < 0.01 (Additional file, Supplementary Table 3) were included in all downstream exploratory analyses. Annotation of these DMPs identified 2,759 unique loci. Most were located within gene bodies (35%) or intergenic regions (IGR: 28%), with fewer in promoter-proximal regions (TSS200: 9%, TSS1500: 14%), the 5′UTR (8%), the first exon (3%), exon boundaries (ExonBnd: 1%), and the 3′UTR (2%) (Supplementary Fig. 2).

**Fig. 1:**
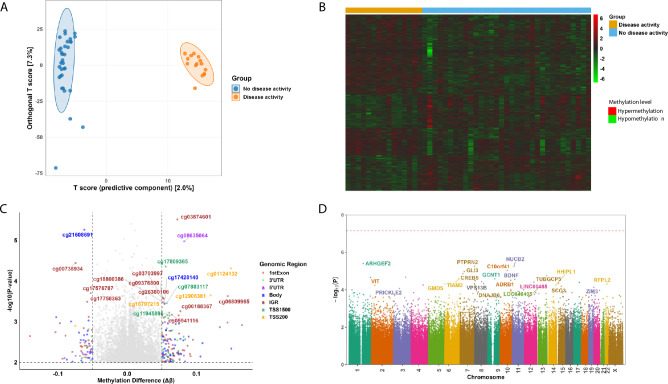
Differential DNA methylation in relation to disease activity in SLE. (**A**) OPLS-DA score plot showing separation of patients based on disease activity status, derived from the top 5,000 most variable CpG sites. (**B**) Heatmap showing methylation levels at significant CpG sites, with red indicating higher methylation and green indicating lower methylation. (**C**) Volcano plot displaying the top 20 differentially methylated CpG sites based on statistical significance and effect size. CpGs with a methylation difference (Δβ) of at least 5% are highlighted. The direction of change is shown relative to the no disease activity group. (**D**) Manhattan plot showing the chromosomal distribution of top DMPs annotated to a known gene. The dashed line represents the Bonferroni-corrected genome-wide significance threshold. IGR, intergenic region; TSS, transcription start site; UTR, untranslated region; ExonBnd, exon boundary.

To explore the pattern of DNA methylation profiles relative to disease activity status, we performed Orthogonal Partial Least Squares Discriminant Analysis (OPLS-DA) using the top 5,000 most variable CpG sites (Fig. 1A). While the OPLS-DA score plot visually separates patients based on disease activity, the predictive component (Q^2^ = 0.030) explains only a small proportion of the variance between groups, despite the overall model capturing a substantial fraction of the total variance (R^2^ = 0.702). The large difference between R^2^ and Q^2^ indicates that the model has limited predictive generalizability and should not be interpreted as a confirmatory predictor of disease activity. This is expected in complex, multifactorial diseases such as SLE, where DNA methylation patterns are subtle and influenced by many biological and non-biological environmental factors. Nevertheless, the model remains meaningful as an exploratory, hypothesis-generating tool, providing insights into patterns in the data and supporting downstream analyses for identifying differentially methylated regions and pathways.

For further exploration, we visualized selected DMPs for hypothesis generation. The heatmap showed distinct methylation patterns between the two groups (Fig. 1B). The top differentially methylated CpG sites, selected based on statistical significance and effect size, are highlighted in the volcano plot (Fig. 1C). The Manhattan plot (Fig. 1D) illustrates the chromosomal distribution of top DMPs annotated to known genes.

**Fig. 2:**
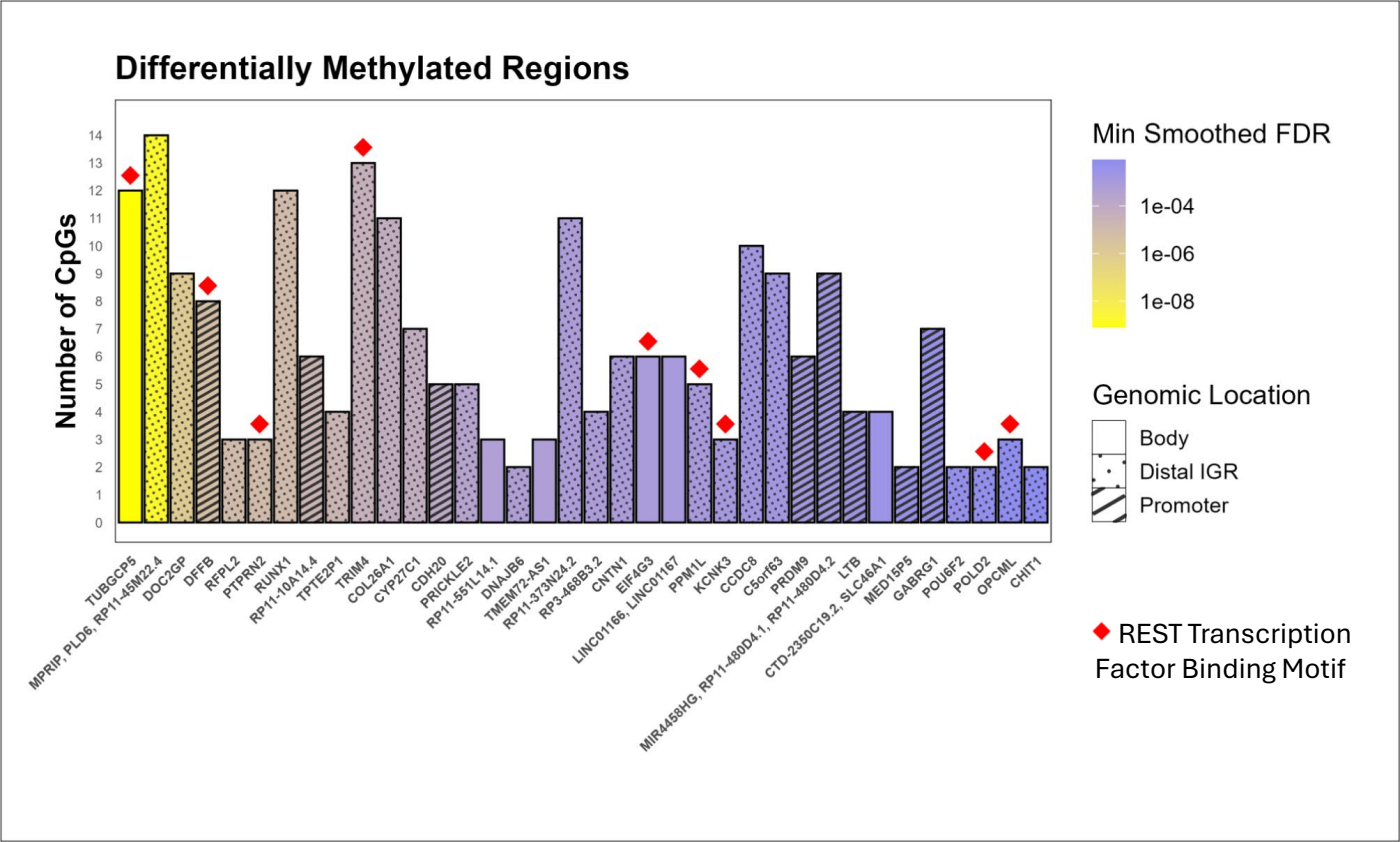
Differentially methylated regions between SLE patients based on disease activity. Bar plot displaying the number of DMRs identified between the two groups, based on smoothed FDR < 0.01. These regions represent contiguous CpG sites showing consistent methylation differences. Filled bar patterns indicate the predominant genomic annotation of CpG sites within each DMR (promoter, gene body, or distal intergenic region). Red diamond symbols denote DMR-associated genes enriched for the REST transcription factor binding motif.

DMR analysis of 4,542 DMPs revealed a total of 36 significant DMRs (min smoothed FDR < 0.01) (Fig. 2, Table 2; see Supplementary Table 5 for additional information). Genomic annotation revealed that the majority of DMRs were located within intronic regions (22/36, 61.1%), followed by promoter regions (8/36, 22.2%; ≤ 3 kb upstream of TSS), and distal intergenic regions (6/36, 16.7%). No DMRs were located within exonic regions. Subsequent motif enrichment analysis of the identified 36 DMRs revealed significant motif enrichment of the REST (RE1-Silencing Transcription factor) binding motif (GGACAGCKC; adjusted *p* = 0.023). Results for all transcription factors tested are provided in Supplementary Table 6.

**Table 2:** Differentially methylated regions.

Overlapping genes	Chromosome	CpG (n)	Min smoothed FDR	Annotation
*TUBGCP5*	chr15	12	7.87E-10	Distal Intergenic
*MPRIP, PLD6, RP11-45M22.4*	chr17	14	1.83E-09	Intron
*DOC2GP*	chr11	9	1.00E-06	Intron
*DFFB*	chr1	8	4.44E-06	Promoter
*RFPL2*	chr22	3	8.51E-06	Intron
*PTPRN2*	chr7	3	9.09E-06	Intron
*RUNX1*	chr21	12	9.09E-06	Intron
*RP11-10A14.4*	chr8	6	2.02E-05	Promoter
*TPTE2P1*	chr13	4	2.02E-05	Intron
*TRIM4*	chr7	13	3.88E-05	Intron
*COL26A1*	chr7	11	6.29E-05	Intron
*CYP27C1*	chr2	7	6.29E-05	Intron
*CDH20*	chr18	5	0.000120083	Promoter
*PRICKLE2*	chr3	5	0.000263379	Intron
*RP11-551L14.1*	chr12	3	0.000488335	Distal Intergenic
*DNAJB6*	chr7	2	0.000549402	Intron
*TMEM72-AS1*	chr10	3	0.000573264	Distal Intergenic
*RP11-373N24.2*	chr6	11	0.000711801	Intron
*RP3-468B3.2*	chr6	4	0.000717507	Intron
*CNTN1*	chr12	6	0.000809152	Intron
*EIF4G3*	chr1	6	0.000873598	Distal Intergenic
*LINC01166, LINC01167*	chr10	6	0.001044559	Distal Intergenic
*PPM1L*	chr3	5	0.001126709	Intron
*KCNK3*	chr2	3	0.001314297	Intron
*CCDC8*	chr19	10	0.001695122	Intron
*C5orf63*	chr5	9	0.001697059	Intron
*PRDM9*	chr5	6	0.001746207	Promoter
*MIR4458HG, RP11-480D4.1, RP11-480D4.2*	chr5	9	0.001950702	Promoter
*LTB*	chr6	4	0.002712462	Promoter
*CTD-2350C19.2, SLC46A1*	chr17	4	0.002879208	Distal Intergenic
*MED15P5*	chr2	2	0.003969657	Promoter
*GABRG1*	chr4	7	0.004315831	Promoter
*POU6F2*	chr7	2	0.005026316	Intron
*POLD2*	chr7	2	0.005779201	Intron
*OPCML*	chr11	3	0.00756808	Intron
*CHIT1*	chr1	2	0.008704201	Intron

**Fig. 3:**
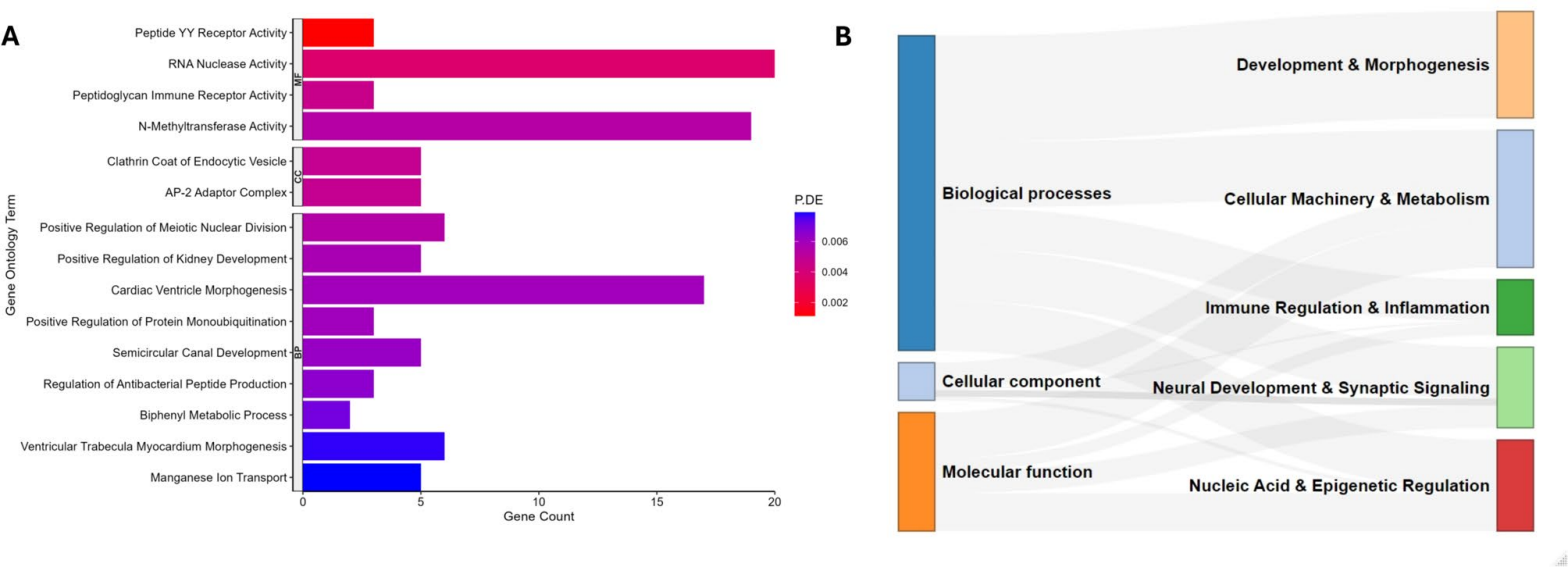
Functional enrichment of differentially methylated positions associated with SLE disease activity. (**A**) Top 15 significantly enriched GO terms associated with disease activity. (**B**) Semantic similarity analysis of enriched GO terms, highlighting relationships among pathways. GO terms are categorized into Molecular Function (MF), Cellular Component (CC), and Biological Process (BP). "P.DE" indicates the *p*-value of enrichment for each term.

Gene Ontology analysis of selected DMPs identified 274 significantly enriched GO terms (Additional file, Supplementary Table 4), with the top 15 most significant terms shown in Fig. 3A. To facilitate biological interpretation, semantic similarity analysis was applied to the enriched GO terms, which grouped them into broader functional categories (Fig. 3B). This clustering revealed five overarching domains: (1) tissue and organ development and morphogenesis, (2) cellular machinery and metabolism, (3) immune regulation and inflammation, (4) neural development and synaptic signaling, and (5) nucleic acid and epigenetic regulation.

## Discussion

This study examined epigenome-wide DNA methylation differences in relation to disease activity in a well-controlled cohort of women with SLE. Participants with evidence of disease activity were younger than those in the no disease activity group, which may reflect variation in disease course across the lifespan. However, all subsequent analyses were adjusted for age, to minimize age-related effects.

No individual CpG site reached statistical significance after correction for multiple testing, which is consistent with a previous report that activity-related methylation differences in SLE are subtle^14^. We therefore adopted a hypothesis-generating, feature-selection strategy at raw p value < 0.01 to explore whether coordinated epigenetic signals could nevertheless be detected at the regional and pathway levels. Using this approach, we identified 4,542 exploratory DMPs, annotated to 2,759 unique loci, suggested widespread but subtle DNA methylation changes in relation to the disease activity. The fact that most DMPs were located within gene bodies and intergenic regions supports previous findings that these genomic elements play key roles in regulating immune cell identity and function in autoimmune diseases^29,30^. While promoter regions are classically associated with transcriptional control, methylation within gene bodies and distal regulatory elements is increasingly recognized for its role in fine-tuning gene expression^31,32^.

Among the top-ranked genes associated with DMPs were *BDNF*, *ARHGEF2*, *CREB5*, and *ADRB1* which are genes implicated in neuroimmune signaling, cellular communication, and inflammation^33–36^. Notably, *BDNF* (Brain-Derived Neurotrophic Factor) has been linked to both neuropsychiatric lupus and immune system modulation^37,38^. Central nervous system involvement, or neuropsychiatric lupus, is frequent in patients with SLE, and includes a wide range of symptoms, from headache, mood disturbances, and mild cognitive impairment to disabling outcomes, such as stroke and seizures^39^. Some of these symptoms, such as mood disorders, cognitive dysfunction and fatigue, are highly prevalent in SLE and remain a significant challenge for patients even when their disease appears to be otherwise well controlled or in remission^4^. Further, immunosuppressive therapy rarely reliably improves these symptoms. Our findings indicate that epigenetic alterations linked to SLE disease activity encompasses domains of neuroinflammation, suggesting that brain-related immune pathways might be altered in those patients with SLE who have well controlled disease but ongoing mild symptoms.

The DMR analysis provided a more robust signal, identifying 36 significant regions (min smoothed FDR < 0.01). Among the top-ranked DMRs, several genes stood out for their relevance to SLE pathogenesis. *RUNX1*, a transcription factor involved in hematopoiesis and lymphocyte development, is critical for T and B cell differentiation and immune tolerance. Its dysregulation has been implicated in autoimmune conditions, and it also influences interferon-stimulated gene expression^40^, a pathway central to SLE pathophysiology. Similarly, TRIM4, an E3 ubiquitin ligase, plays a regulatory role in innate immunity and type I interferon signaling, an axis consistently overactive in SLE^41^. Altered methylation of *TRIM4* may reflect the aberrant regulation of antiviral and immune responses contributing to systemic inflammation. *DFFB*, which encodes a key endonuclease that mediates DNA fragmentation during apoptosis, is also noteworthy. Defective clearance of apoptotic cells is a well-documented trigger for SLE autoimmunity due to the accumulation of nuclear antigens^42^. Epigenetic regulation of *DFFB* could impair apoptotic cell processing, promoting autoantigen exposure and sustained immune activation^43^. PTPRN2, although less studied in SLE, is involved in neuroendocrine secretion and has been identified as an autoantigen in other autoimmune conditions^44,45^, raising the possibility of its role as a novel autoantigen in SLE. LTB (lymphotoxin beta) is a member of the TNF superfamily, involved in lymphoid organogenesis, inflammation, and cytokine signaling^46,47^. It has been linked to chronic inflammatory signaling and has been found upregulated in lupus-related inflammation^48^.

In addition to immune-related genes, a subset of DMR-associated genes was annotated to neuronal and synaptic functions, including *CNTN1*, *POU6F2*, *PRICKLE2*, *DNAJB6*, GABRG1 and *OPCML*. CNTN1, a neuronal adhesion molecule, is a target of autoantibodies in neuroimmune disorders and may be implicated in neuropsychiatric lupus^49,50^. POU6F2 and PRICKLE2, involved in neuronal development and polarity, are linked to seizure disorders and autism, conditions with overlapping features in neuropsychiatric SLE^51,52^. DNAJB6, is a molecular chaperone associated with neural proteostasis, which has been implicated in neurodegenerative diseases^53^. GABRG1 is a subunit of the GABA-A receptor, critical for inhibitory neurotransmission in the brain^54^. Altered GABAergic signaling is associated with anxiety, seizures, and cognitive dysfunction^55^, which are seen in neuropsychiatric SLE , provides a neurochemical link to SLE-related cognitive and psychiatric symptoms. Similarly, OPCML, a GPI-anchored neural adhesion protein, has roles in synaptic communication^56^, potentially disrupted in SLE patients with cognitive or psychiatric symptoms. In SLE, epigenetic modulation of such genes in disease activity could reflect neural stress, injury, or systemic neural-immune interaction. However, these observations should be interpreted cautiously as they were derived from whole blood and lack corresponding transcriptomic or chromatin accessibility measurements, therefore, we cannot infer whether these loci are directly involved in neuropsychiatric manifestations of SLE. Rather, these results indicate that epigenetic differences associated with disease activity are enriched in genes with known roles in both immune and neural biology, which is consistent with the growing recognition of immune–nervous system crosstalk in autoimmune disease.

In addition, other top-ranked DMRs such as *TUBGCP5*, *MPRIP*, and *DOC2GP* may point toward broader cellular processes that could contribute to disease pathophysiology. *TUBGCP5* encodes a component of the γ-tubulin ring complex involved in microtubule nucleation and cytoskeletal organization^57^. Cytoskeletal dynamics are essential for immune cell migration, immune synapse formation, and intracellular trafficking, processes that are frequently dysregulated in autoimmune disease^58^. MPRIP (myosin phosphatase Rho interacting protein) regulates actin cytoskeleton remodeling through the RhoA signaling pathway, which influences leukocyte motility, adhesion, and endothelial interactions^59^. Epigenetic alterations affecting cytoskeletal regulators could therefore impact immune cell trafficking and tissue infiltration in active SLE. *DOC2GP*, a gene related to calcium-dependent vesicle trafficking proteins^60^, may reflect altered intracellular signaling or secretory processes, although its functional relevance in immune cells remains poorly characterized. While these genes have not been directly implicated in SLE to date, their involvement in cytoskeletal organization, cell signaling, and vesicular dynamics suggests that disease-associated methylation changes may extend beyond canonical immune pathways and affect fundamental cellular mechanisms that shape immune cell activation and function.

Our results show also enrichment of the REST (RE1-Silencing Transcription Factor) binding motif within the DMRs. REST is a transcriptional repressor mostly involved in neuronal gene expression but also in immune responses^61–63^. Its enrichment among DMRs might indicate dysregulated REST activity in SLE, potentially leading to inappropriate activation of neuronal genes in leukocytes, where such genes are normally silent, alongside immune gene expression. However, motif enrichment indicates sequence compatibility with REST binding, not functional occupancy. Without ChIP-seq or chromatin accessibility data, this finding should serve as a basis for generating hypothesis rather than as evidence of active REST-mediated regulation in SLE.

Taken together, these results suggest that subtle, coordinated epigenetic differences exist between clinically quiescent and mildly active SLE, detectable primarily at the regional and pathway level. These patterns point to candidate immune and neuro-annotated pathways that merit validation in larger, longitudinal, and cell-type–specific studies.

## Limitations

Several limitations of this study should be acknowledged. First, the relatively small sample size limited statistical power, and the absence of an independent replication cohort affected the robustness and generalizability of the findings. In addition, because no CpG sites reached the FDR threshold for definitive significance, technical validation using targeted approaches such as pyrosequencing was not performed. Replication in larger, independent cohorts will be necessary to confirm these results, and the findings should be interpreted as exploratory and hypothesis-generating rather than confirmatory. Second, due to the cross-sectional study design, temporal relationships, causality, and biomarker utility cannot be inferred, nor changes in DNA methylation over time in relation to disease activity can be assessed.

Third, DNA methylation was assessed in whole blood, which introduces cell-type heterogeneity and may obscure cell type–specific epigenetic alterations. Although immune cell proportions were estimated and adjusted for, these values represent computational inferences rather than direct measurements. As a result, it cannot be definitively determined whether the observed methylation differences reflect true within–cell-type epigenetic changes or shifts in underlying immune cell population proportions associated with disease activity. Future studies using sorted immune cell populations or single-cell methylomic approaches will be necessary to provide greater cellular resolution and mechanistic insight. Fourth, the cohort exclusively included female participants, limiting generalizability for male patients with SLE. Given known sex differences in epigenetic regulation and immune responses, inclusion of male participants in future studies will be important to assess the broader applicability of these findings (C^64,65,66^). In addition, although most participants exhibited relatively low disease activity, grouping all individuals with SLEDAI-2K > 0 into a single “active” category introduces heterogeneity that may dilute methylation signals associated with specific levels of disease severity. Larger cohorts encompassing a broader range of disease activity, stratified into smaller groups, will therefore be necessary to more precisely characterize epigenetic changes across the SLE disease spectrum. Lastly, immunosuppressive therapy represents a potential confounder, as treatment exposure influenced methylation patterns in sensitivity analyses. Due to the small number of untreated patients, therapy could not be robustly modeled without risk of overfitting, and medication effects may therefore contribute to the observed differences.

## Conclusions

In summary, this study provides exploratory evidence suggesting that differences in DNA methylation, particularly in genes and pathways related to immune function and neural regulation, may be associated with disease activity in women with SLE. While single-site signals did not survive FDR correction, region-level analyses showed convergent DMRs, mapping to immune and neuroimmune pathways. Moreover, our results also show an enrichment of the REST binding motif in disease activity, involved in the regulation of neuronal and immune gene expression. This work highlights the potential relevance of epigenetic changes to disease activity and suggests novel molecular pathways involved in SLE. Validation in larger, longitudinal, cell-type–resolved cohorts is needed to confirm clinical utility and clarify mechanism.

## Supplementary Information

Supplementary Information 1.

Supplementary Information 2.

Supplementary Information 3.

## Data Availability

All study data are available from the main author of the manuscript upon reasonable request.

## Abbreviations

ADRB1: Beta-1 adrenergic receptor
ARHGEF2: Rho guanine nucleotide exchange factor 2
BDNF: Brain-derived neurotrophic factor
BMI: Body mass index
BMIQ: Beta mixture quantile
bDMARD: Biologic disease-modifying anti-rheumatic drug
CpG: Cytosine-phosphate-guanine dinucleotide
CREB5: CAMP Response element-binding protein 5
CRP: C-reactive protein
csDMARD: Conventional synthetic disease-modifying anti-rheumatic drug
DFFB: DNA fragmentation factor subunit beta
DMARD: Disease-modifying anti-rheumatic drug
DMPs: Differentially methylated positions
DMRs: Differentially methylated regions
DNAJB6: DnaJ heat shock protein family member B6
DOC2B: Double C2-like domain-containing protein beta
dsDNA: Double-stranded DNA
EQ-5D: EuroQol 5-dimension score
ESR: Erythrocyte sedimentation rate
FDR: False discovery rate
GABRG1: Gamma-aminobutyric acid type a receptor subunit gamma1
GO: Gene ontology
HAQ: Health assessment questionnaire
LTB: Lymphotoxin beta
MAF: Minor allele frequency
MPRIP: Myosin phosphatase rho interacting protein
NSAIDs: Non-steroidal anti-inflammatory drugs
OPCML: Opioid binding protein/cell adhesion molecule-like
OPLS-DA: Orthogonal partial least squares discriminant analysis
POU6F2: POU class 6 homeobox 2
RPC: Robust partial correlations
PRICKLE2: Prickle planar cell polarity protein 2
PTPRN2: Protein tyrosine phosphatase receptor type N2
REST: RE1-silencing transcription factor
RUNX1: Runt-related transcription factor 1
SLE: Systemic lupus erythematosus
SLEDAI-2 K: Systemic lupus erythematosus disease activity index 2000
SLICC: Systemic lupus international collaborating clinics damage index
SS-A/Ro: Sjögren’s-syndrome-related antigen A
SS-B/La: Sjögren’s-syndrome-related antigen B
TRIM4: Tripartite motif containing 4
TUBGDP5: Tubulin gamma complex component 5
