# Supplementary material for: Epigenome-wide DNA methylation patterns associated with disease activity in systemic lupus erythematosus
Source: Sci Rep. 2026 May 5;16:14287. doi: 10.1038/s41598-026-51708-3 (PMC13144499; doi:10.1038/s41598-026-51708-3)
Supplement: Supplementary file 2 — Supplementary Information 2. [file 41598_2026_51708_MOESM2_ESM.pdf]

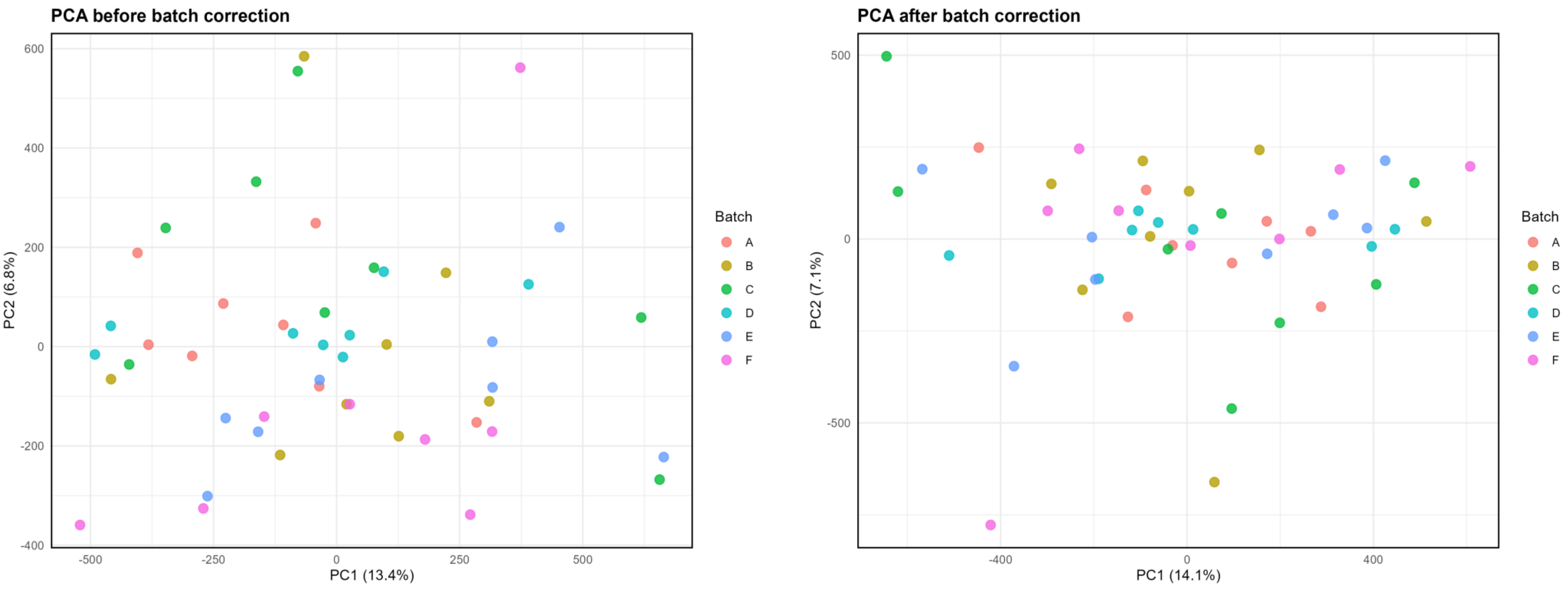

Supplementary Figure 1. Principal component analysis (PCA) of DNA methylation profiles before (left) and after (right) batch correction. Batch effects were accounted for by including batch as a covariate in the limma linear model. Following correction, batch-driven clustering is reduced, indicating effective mitigation of technical variation.
