# Supplementary material for: Epigenome-wide DNA methylation patterns associated with disease activity in systemic lupus erythematosus
Source: Sci Rep. 2026 May 5;16:14287. doi: 10.1038/s41598-026-51708-3 (PMC13144499; doi:10.1038/s41598-026-51708-3)
Supplement: Supplementary file 3 — Supplementary Information 3. [file 41598_2026_51708_MOESM3_ESM.pdf]

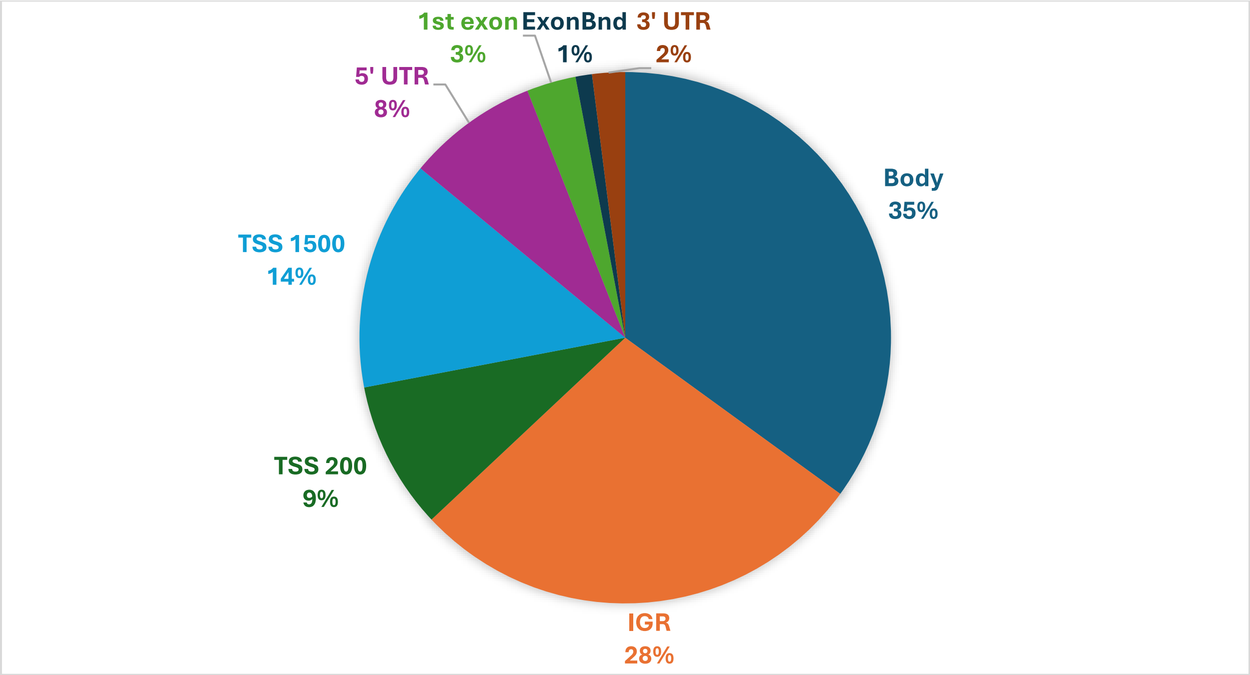

Supplementary Figure 2. Genomic distribution of differentially methylated positions (DMPs). A total of 4,542 DMPs were identified across 48 patients. Most loci were located in gene bodies and intergenic regions, with smaller proportions in promoter-proximal and untranslated regions.
